# Immune System and Chronic Diseases 2018

**DOI:** 10.1155/2018/8653572

**Published:** 2018-10-24

**Authors:** Margarete Dulce Bagatini, Andréia Machado Cardoso, Cristina Ruedell Reschke, Fabiano Barbosa Carvalho

**Affiliations:** ^1^Coordenação Acadêmica, Universidade Federal da Fronteira Sul, Campus Chapecó, Chapecó, SC, Brazil; ^2^Department of Physiology and Medical Physics, Royal College of Surgeons in Ireland, Dublin D02 YN77, Ireland; ^3^Laboratório de Pesquisa em Patologia, Universidade Federal de Ciências da Saúde de Porto Alegre, Porto Alegre, RS, Brazil

The immune system has a central role in many processes involving chronic diseases. The recognition of altered immune system function in many chronic disease states has proven to be a pivotal advance in biomedical research over the past decade. For many metabolic disorders, this altered immune activity has been characterized as inflammation. However, accumulating evidence challenges this assumption and suggests that the immune system may be mounting adaptive responses to chronic stressors. Immunological research involving cancer, rheumatoid arthritis, inflammatory bowel disease, asthma, multiple sclerosis diabetes, heart diseases, and others has not only enabled the understanding of the mechanisms that underlie these diseases but also suggested new therapies that may impact positively on patients minimizing morbidity and mortality. This special issue is aimed at presenting and discussing the advancement of research and innovative therapies involving chronic diseases, which have a high impact on society.

Evidences show that purinergic signaling is involved in processes associated with the immune system in health and disease, including noncommunicable, neurological, and degenerative diseases. These diseases strike from children to elderly and are generally characterized by progressive deterioration of cells, eventually leading to tissue or organ degeneration. These pathological conditions can be associated with disturbance in the signaling mediated by nucleotides and nucleosides of adenine, in expression or activity of extracellular ectonucleotidases, and in activation of P2X and P2Y receptors. Among these diseases, the best known are atherosclerosis, hypertension, cancer, epilepsy, Alzheimer's disease (AD), Parkinson's disease (PD), and multiple sclerosis (MS). The currently available treatments present limited effectiveness and are mostly palliative. M. D. Bagatini and collaborators reviewed the role of purinergic signaling highlighting the ectonucleotidases E-NTPDase, E-NPP, E-5-nucleotidase, and adenosine deaminase in noncommunicable, neurological, and degenerative diseases associated with the cardiovascular and central nervous systems and cancer.

Considering the involvement of the immune system in cancer, Y. Matsukiyo and colleagues conducted a clinical study that evaluated host immunity in patients with cirrhosis receiving partial splenic embolization (PSE) for thrombocytopenia. Restoration of the balance between T lymphocyte subsets and Th1/Th2 cytokines followed by an improvement of antitumor immunity has been reported after hepatosplenectomy in patients with liver cirrhosis and hepatocellular carcinoma. In fact, after PSE, an increase in counts for platelets, neutrophils, lymphocytes, and monocytes was verified. In parallel, Th1 and Th2 as well as TNF-alpha, TNF receptor I, and soluble Fas showed a significant increase at 4 weeks after PSE. These evidences found by Y. Matsukiyo and collaborators indicate that PSE can recover leukopenia and thrombocytopenia in patients with cirrhosis and thrombocytopenia.

In this line, H. G. Dursun and colleagues studied the association of cytotoxic T lymphocyte antigen-4 gene polymorphisms with psoriasis vulgaris in Turkish population and determined whether CTLA-4 gene polymorphisms are associated with the development and/or clinical features of psoriasis vulgaris. Psoriasis is a common, chronic, and autoimmune skin disease in which dysregulation of immune cells, particularly T cells, is thought to play an important role in the pathogenesis. Cytotoxic T lymphocyte antigen-4 (CTLA-4) expressed only on activated T cells is an immunoregulatory molecule and plays a role in the pathogenesis of autoimmune disorders. In the same disease, S. Cohen et al. found high expression levels of the Gi protein-associated A3 adenosine receptor (A3AR) and explored its role in mediating the anti-inflammatory effect of piclidenoson, a selective agonist at the A3AR.

Inflammation is the central driving force in much of these chronic degenerative diseases. Noncommunicable inflammatory diseases affect nearly all organ systems of the body including the skin, endocrine glands, gut, lungs, kidneys, and musculoskeletal and cardiovascular system. S. Schröder et al. investigated the modulation of inflammatory reactions after low-dose radiotherapy (LD-RT) on the basis of endothelial cells (EC) and showed that LD-RT, also using very low radiation doses, has a clear immunomodulatory effect on EC as major participants and regulators of inflammation.

Rheumatoid arthritis (RA) is an often debilitating autoinflammatory disease, and patients with this pathology commonly present psychological symptoms. From this, M. Figueiredo-Braga and colleagues carried out a cohort study in RA patients with the purpose of verifying the influence of the levels of cytokines and their inhibitors on psychological symptoms more frequently described. Depression, anxiety, sleep, fatigue, and relationship status were the psychological symptoms investigated and associated with levels of cytokines. They have verified that IL-10 and IL-6 are likely to be involved with depressive symptoms. IL-10 concentration was associated with depression, and tocilizumab decreased depressive symptoms in the RA patients. In parallel, IL-6 was also associated with depressive symptoms in patients with primary depression. These findings contributed significantly to understanding possible mechanisms that trigger psychological symptoms commonly found in RA. In addition, the study highlights that cytokine levels have a strong impact on depressive symptoms.

R. A. Iseme et al. studied the association between autoantibodies and qualitative ultrasound index of bone in an elderly sample without clinical autoimmune disease. Bone loss is characteristic of the ageing process and a common complication of many autoimmune diseases. Research has highlighted a potential role of autoantibodies in pathologic bone loss. In this line, F. M. Perrotta et al. investigated the serum levels of sclerostin in patients with ankylosing spondylitis (AS) as a possible biomarker and for investigating any correlations with radiographic damage, disease activity, and function, considering that several molecules are involved in the pathogenesis of a new bone formation.

M. R. Barros Jr. et al. reviewed the HPV immune evasion mechanisms involving Toll-like receptors (TLRs) and cytokines and discussed the existing and potential immunotherapeutic TLR- and cytokine-related tools. The authors revealed that HPV-related tumors require a great immune suppressor status for cancer development with increased activities of Treg, CTLA-4, and PD-1 and the suppression of APC and NK cells and that the therapies should consider the tumor-related evasion mechanisms. H.W. Grievink and M. Moerland speculated that sample aging induces an inhibitory pathway downstream from Toll-like receptor 4 in monocytes. These results underline the importance of quick sample handling when investigating innate immune responses in whole blood, especially for monocyte responses. S. Wang et al. evaluated the protective effects of sublancin on immunosuppression in cyclophosphamide-treated mice and suggested that sublancin plays a crucial role in the protection against immunosuppression in cyclophosphamide-treated mice and could be a potential candidate for immune therapy regimens.

A. Puccetti et al. identified specific miRNA signatures associated with Behçet's disease (BD) patients with active disease and concluded that the combined analysis of deregulated miRNAs and BD transcriptome sheds light on some epigenetic aspects of BD identifying specific miRNAs, which may represent promising candidates as biomarkers and/or for the design of novel therapeutic strategies in BD. R. A. G. Khammissa et al. reviewed the clinical and histopathological aspects of adverse immunologically mediated oral mucosal reactions to systemic medication, which seems to be occurring in genetic susceptible patients.

Bronchial asthma is an important cause of chronic morbidity affecting children and adults worldwide. In this pathology, eosinophil infiltration releases a number of cationic proteins, including the major basic protein (MBP) and the eosinophil cationic protein (ECP), which can lead to the damage of the bronchial epithelial cells. To understand the role of cationic proteins in bronchial epithelial cells, Y.-N. Wang and colleagues conducted an experimental investigation to find out whether poly-L-arginine (PLA), a synthetic cationic protein, induces apoptosis in the human airway epithelial cells (NCI-H292) as well as cellular targets involved in this effect. In fact, PLA triggered apoptosis in NCI-H292 cells and altered the signaling pathways ERK1/2 and Bcl2/Bax. These evidences point to the inhibition of cationic protein as a potential target of anti-injury or antiremodeling in asthmatics.

